# Response and resistance to cladribine in patients with advanced systemic mastocytosis: a registry-based analysis

**DOI:** 10.1007/s00277-023-05180-y

**Published:** 2023-04-04

**Authors:** Johannes Lübke, Nicole Naumann, Georgia Metzgeroth, Sebastian Kreil, Timo Brand, Hans-Peter Horny, Karl Sotlar, Nicholas C. P. Cross, Alice Fabarius, Peter Valent, Wolf-Karsten Hofmann, Andreas Reiter, Juliana Schwaab

**Affiliations:** 1 Department of Hematology and Oncology, University Hospital Mannheim, Heidelberg University, Mannheim, Germany; 2 Department of Pathology, Ludwig-Maximilians-University, Munich, Germany; 3 Department of Pathology, Paracelsus Medical University of Salzburg, Salzburg, Austria; 4 Faculty of Medicine, University of Southampton, Southampton, UK; 5 Department of Internal Medicine I, Division of Hematology & Hemostaseology, Medical University of Vienna, Vienna, Austria; 6 Ludwig Boltzmann Institute for Hematology and Oncology, Medical University of Vienna, Vienna, Austria

**Keywords:** Advanced systemic mastocytosis, Cladribine, Chemotherapy, Purine analogue

## Abstract

We sought to evaluate the efficacy of the purine analogue cladribine in 79 patients with advanced systemic mastocytosis (AdvSM) using data from the ‘German Registry on Disorders of Eosinophils and Mast Cells (GREM)’. The overall response rate according to modified Valent criteria (46 evaluable patients) for first- (1L) and second-line (2L) cladribine treatment was 41% (12/29) and 35% (6/17, *P* = 0.690), respectively, and the median overall survival (OS, all patients evaluable) was 1.9 years (*n* = 48) and 1.2 years (*n* = 31; *P* = 0.311). Univariate and multivariable analyses of baseline and on-treatment parameters identified diagnosis of mast cell leukemia (hazard ratio [HR] 3.5, 95% confidence interval [CI, 1.3–9.1], *P* = 0.012), eosinophilia ≥ 1.5 × 10^9^/L (HR 2.9 [CI 1.4–6.2], *P* = 0.006) and < 3 cycles of cladribine (HR 0.4 [CI 0.2–0.8], *P* = 0.008) as independent adverse prognostic parameters for OS. There was no impact of other laboratory (anemia, thrombocytopenia, serum tryptase) or genetic markers (mutations in *SRSF2*, *ASXL1* or *RUNX1*) on OS. In consequence, none of the recently established prognostic scoring systems (MARS, IPSM, MAPS or GPSM) was predictive for OS. Modified Valent criteria were superior to a single factor-based response assessment (HR 2.9 [CI 1.3–6.6], *P* = 0.026). In conclusion, cladribine is effective in 1L and 2L treatment of AdvSM. Mast cell leukemia, eosinophilia, application of < 3 cycles and a lack of response are adverse prognostic markers.

**Supplementary Information:**

The online version contains supplementary material available at 10.1007/s00277-023-05180-y.

## Introduction

Systemic mastocytosis (SM) is a rare myeloid neoplasm characterized by multifocal accumulation of neoplastic mast cells (MC) in the bone marrow (BM), visceral organs and skin [1–4]. Advanced systemic mastocytosis (AdvSM) comprises aggressive SM (ASM), SM with an associated hematologic neoplasm (AHN), and MC leukemia (MCL). SM phenotype driver is an acquired somatic point mutation in *KIT* at codon D816V (*KIT* D816V) found in > 90% of AdvSM patients [5, 6]. In addition, 60–80% of patients harbor additional somatic mutations, e.g. in *SRSF2*, *ASXL1*, *RUNX1* (*S/A/R* gene panel), *NRAS*, or *DNMT3A*, which are important parameters for combined clinico-genetic prognostic risk scoring systems (e.g., Mutation-Adjusted Risk Score, MARS; Mayo Alliance Prognostic System, MAPS; Global Prognostic Score for SM, GPSM) [7–12].

The development of novel targeted drugs, e.g., the multikinase inhibitor midostaurin [13–15] and the *KIT* D816V inhibitor avapritinib [16, 17], has extended the therapeutic options for patients with AdvSM, which were previously based on the off-label use of the purine analogue cladribine [18–22]. However, recent data on response rates and variably on leukemia-free (LFS), event-free- (EFS) and overall survival (OS) meanwhile favor the use of midostaurin and avapritinib [23–26]. Notwithstanding, cladribine will remain a relevant treatment option beyond first-line treatment due to intolerance, resistance and progression on KIT inhibitors [23, 27, 28]. No predictive markers have yet been established for response, resistance and survival in cladribine-treated AdvSM patients [18–22], a gap which we aimed to fill by analysis of a comprehensive cohort of 79 cladribine-treated patients enrolled within the ‘German Registry on Disorders of Eosinophils and Mast Cells’ (GREM).

## Patients and methods

### Study population

All cladribine-treated patients (*n* = 79) from the GREM which were diagnosed between 2003 and 2021 were selected for this project, which is an updated and more detailed analysis of a comparative study between midostaurin and cladribine [23]. The diagnosis of SM was established according to the World Health Organization classification [1, 29–31]. All BM biopsies were evaluated by reference pathologists (H.-P.H., K.S.) of the European Competence Network on Mastocytosis (ECNM) [32]. The study design adhered to the tenets of the Declaration of Helsinki and was approved by the institutional review board of the Medical Faculty of Mannheim, Heidelberg University, Germany. Written informed consent was provided by all patients.

### Treatment

The number of patients allowed separation of first- (1L) and second-line (2L) treatment. Prior treatment included midostaurin while subsequent treatment approaches included (individually or sequentially) midostaurin, avapritinib, acute myeloid leukemia-like intensive chemotherapy and, rarely, allogeneic stem cell transplantation. Treatment options with a potentially low disease-modifying impact (e.g. interferon-alpha) or solely directed towards AHN (e.g. hydroxyurea, azacytidine) were not considered as 1L- or 2L-treatment.

### Gene mutation analyses

Quantitative assessment of the *KIT* D816V expressed allele burden (EAB) was performed by allele-specific quantitative real-time reverse-transcriptase polymerase chain reaction (RT-qPCR) analysis on RNA/complementary DNA as previously described [33]. NGS analyses on DNA were performed through library preparation by the Access Array Technology (Fluidigm, San Francisco, CA) and sequencing on the MiSeq Instrument (Illumina, San Diego, CA). Gene mutations were annotated using the reference sequence of the Ensembl Transcript ID (Ensembl release 85: July 2016).

### Prognostic scoring systems

The predictive value and clinical utility of several recently established prognostic scoring systems (MARS, International Prognostic Scoring System for AdvSM [IPSM-AdvSM], MAPS, and GPSM) was conducted according to published criteria [7, 11, 12, 34]. Similarities and differences between the scores are given elsewhere. [11, 30]

### Response assessment

Response assessment according to modified Valent criteria [21] included regular monitoring of C-findings, serum tryptase and a BM biopsy within 2 months after the last applied course of cladribine. The reasons for not using the more recently established International Working Group-Myeloproliferative Neoplasms Research Treatment-ECNM (IWG-MRT-ECNM) criteria included: (i) the retrospective nature of our analysis did not allow to adequately address the complex IWG-MRT-ECNM criteria, (ii) the modified Valent response criteria were commonly used for response assessment of cladribine in prior studies. Molecular response was defined as *KIT* D816V expressed allele burden reduction ≥ 25% within 2 months after the last course. [7, 23, 33, 35]

### Statistical analyses

All statistical analyses considering clinical, laboratory and molecular parameters were obtained at the time of diagnosis/first referral to our center (initial parameters), treatment initiation with cladribine (baseline parameters) and at multiple time points during treatment (including time point for response assessment). The Mann–Whitney *U*-test was used to compare continuous variables and medians of distributions. Fisher’s exact test was used for categorical variables. We retrospectively analyzed the OS (time of diagnosis/treatment initiation to the date of death/last visit) by using the Kaplan–Meier method with log-rank test for group comparisons/visualizations. Disease progression was defined as a shift to a more aggressive AdvSM subtype (secondary MCL or secondary acute myeloid leukemia [AML]). Duration of treatment was defined as the duration from initiation of cladribine to discontinuation for any reason. For the estimation of hazard ratios (HRs) and multivariable analysis, the Cox proportional hazard regression model was used. All variables that showed prognostic significance in univariate analyses were included in multivariable analyses. The first multivariable analysis was performed in an unmodified cohort of patients irrespective of prior or following treatment approaches (midostaurin, avapritinib, intensive chemotherapy and allogeneic stem cell transplantation); the second multivariable analysis was performed in a modified cohort in which patients with prior or following treatment approaches were either excluded or censored at the time of initiation of the next treatment line. *P* values of < 0.05 (two-sided) were considered as significant. Data management and statistical analyses were performed with SPSS (SPSS version 20.0; IBM Corporation, Armonk, NY) and GraphPad Prism software (version 8, GraphPad, La Jolla, CA, USA).

## Results

### Therapeutic modalities

Cladribine was used at a dose of 0.14 mg/kg/day subcutaneously or intravenously on days 1–5 of a 28-day course. For both 1L- (*n* = 48, 61%) and 2L-treatment (*n* = 31, 39%), a median number of 3 cycles (range 1–6 and 1–8, respectively) was applied over a median of 3.3 (range 0.1–16.0) and 3.0 months (range 0.1–28.5), respectively (*P* = 0.612; Table 1). Three or more cycles were applied in 32/79 (41%) patients (1L, *n* = 21, 44%; 2L, *n* = 11, 35%). The main reasons for dose reduction, e.g. application only on days 1–3 or extension of intervals, was prolonged myelosuppression (15/79, 19%).

**Table 1:** Demographic and disease characteristics of 79 cladribine treated stratified according first- and second-line treatment

	All	First-line	Second-line	*P*
Number of patients at baseline, *n* (%)	79	48 (61)	31 (39)	
Age in years at treatment initiation; median (range)	68 (27–87)	69 (27–81)	66 (48–87)	0.770
Male, *n* (%)	53 (79)	32 (48)	21 (68)	0.921
Diagnosis				
ASM, *n* (%)	9 (11)	7 (15)	2 (7)	0.267
SM-AHN, *n* (%)	56 (71)	35 (73)	21 (68)	0.621
MCL ± AHN, *n* (%)	14 (18)	6 (13)	8 (26)	0.130
C-findings				
Hemoglobin, g/dL; median (range)	10 (7–15)	11 (7–13)	9 (7–15)	0.124
Platelets, × 10^9^/L; median (range)	99 (12–630)	105 (12–630)	87 (25–388)	0.254
ANC, × 10^9^/L; median (range)	5 (0–65)	6 (1–65)	4 (0–62)	0.648
Alkaline phosphatase, U/L; median (range)	270 (45–1736)	242 (45–1736)	300 (63–919)	0.580
Albumin level, g/L; median (range)	34 (15–48)	34 (21–44)	34 (15–48)	0.709
Other relevant parameters				
Leukocytes, × 10^9^/L; median (range)	9.8 (1.3–14.2)	10.4 (1.3–10.4)	9.0 (2.6–14.2)	0.799
Monocytes, × 10^9^/L; median (range)	0.9 (0.0–18.5)	1.1 (0.0–17.9)	0.9 (0–18.5)	0.862
Eosinophils, × 10^9^/L; median (range)	0.5 (0.0–68.3)	0.5 (0.0–1.4)	0.3 (0.0–68.3)	0.254
MC-infiltration in BM biopsy, %; median (range)	45 (3–100)	40 (5–100)	58 (3–90)	0.023
Serum tryptase level, µg/L; median (range)	215 (23–1200)	199 (23–1150)	448 (54–1200)	0.018
Splenomegaly, *n* (%)	64 (94)	41 (91)	23 (100)	0.141
*KIT* D816V EAB in PB, %, median (range)	35 (0–80)	35 (0–61)	37 (0–80)	0.409
MARS score at diagnosis, *n* (%)	69 (87)	40 (83)	29 (94)	
Low-risk, *n* (%)	16 (23)	10 (25)	6 (21)	0.675
Intermediate-risk, *n* (%)	11 (16)	6 (15)	5 (17)	0.802
High-risk, *n* (%)	42 (61)	24 (60)	18 (62)	0.862
Treatment and outcome				
Follow-up, years since diagnosis; median (range)	2.5 (0.1–17.0)	2.6 (0.1–17.0)	2.2 (0.1–16.4)	0.821
Follow-up, years since 1^st^ cycle; median (range)	1.2 (0.0–12.0)	1.5 (0.0–12-0)	0.8 (0.0–9.6)	0.186
Years to treatment since diagnosis; median (range)	0.7 (0.0–11.0)	0.5 (0.0–10.1)	1.0 (0.1–8.8)	0.083
Years of treatment duration; median (range)	0.3 (0.0–2.4)	0.3 (0.0–1.3)	0.3 (0.0–2.4)	0.612
Number of cladribine cycles, median (range)	3 (1–8)	3 (1–6)	3 (1–8)	0.743
Cycles per months, median (range)	1.0 (0.4–4.8)	1.0 (0.4–4.0)	1.0 (0.7–4.8)	0.848
Deaths, *n* (%)	53 (67)	34 (71)	19 (61)	0.378
Median OS, years (95% CI)	1.5 (1.0–2.0)	1.9 (1.1–2.6)	1.2 (0.3–2.1)	0.311

*ANC* Absolute neutrophil count; *ASM* Aggressive systemic mastocytosis; *BM* Bone marrow; *CI* Confidence interval; *EAB* Expressed allele burden; *MARS* Mutation-adjusted risk score; *MC* Mast cell; *MCL* ± *AHN* Mast cell leukemia with/without an associated hematologic neoplasm; *NR* Monocytosis non-response; *OS* Overall survival; *PB* Peripheral blood; *R* Monocytosis response; *SM-AHN* Systemic mastocytosis with an associated hematological neoplasm

An expanded version of this table is given as Appendix Table 2

### Comparison of baseline characteristics

Compared to 1L-treatment, patients on 2L-treatment presented with a higher frequency of anemia (61% vs. 35%,* P* = 0.039), a higher percentage of BM MC infiltration (58% vs. 40%, *P* = 0.023) and a higher median serum tryptase level (448 vs. 199 µg/L, *P* = 0.018). No significant differences were observed regarding median time from diagnosis (2.2 vs. 2.6 years, *P* = 0.821) and median time from start of treatment (0.8 vs. 1.5 years, *P* = 0.186; Table 1, Appendix Table 2).

### Evaluation of on-treatment and outcome parameters

**Fig. 1:**
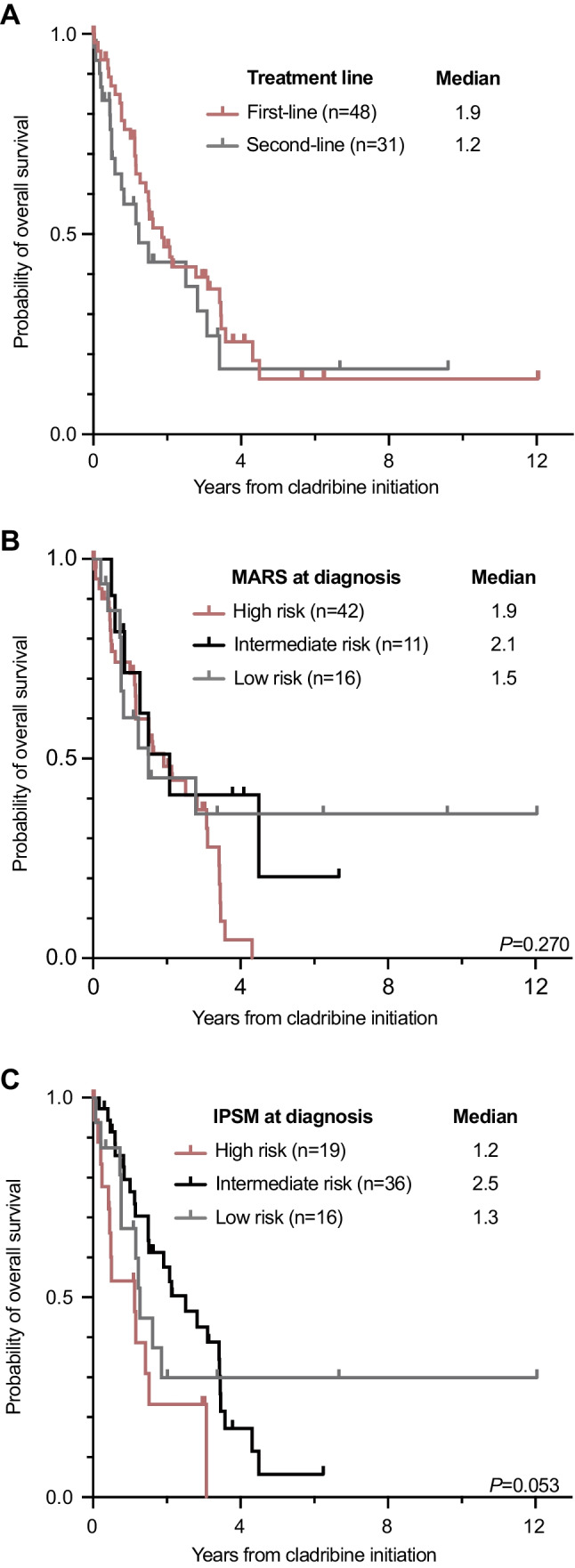
**(A)** Kaplan-Meier estimates of overall survival in cladribine treated patients stratified according to the modified Valent response categories. Respective analyses were performed for cladribine in first- **(B)** and second-line **(C)** use

**Fig. 2:**
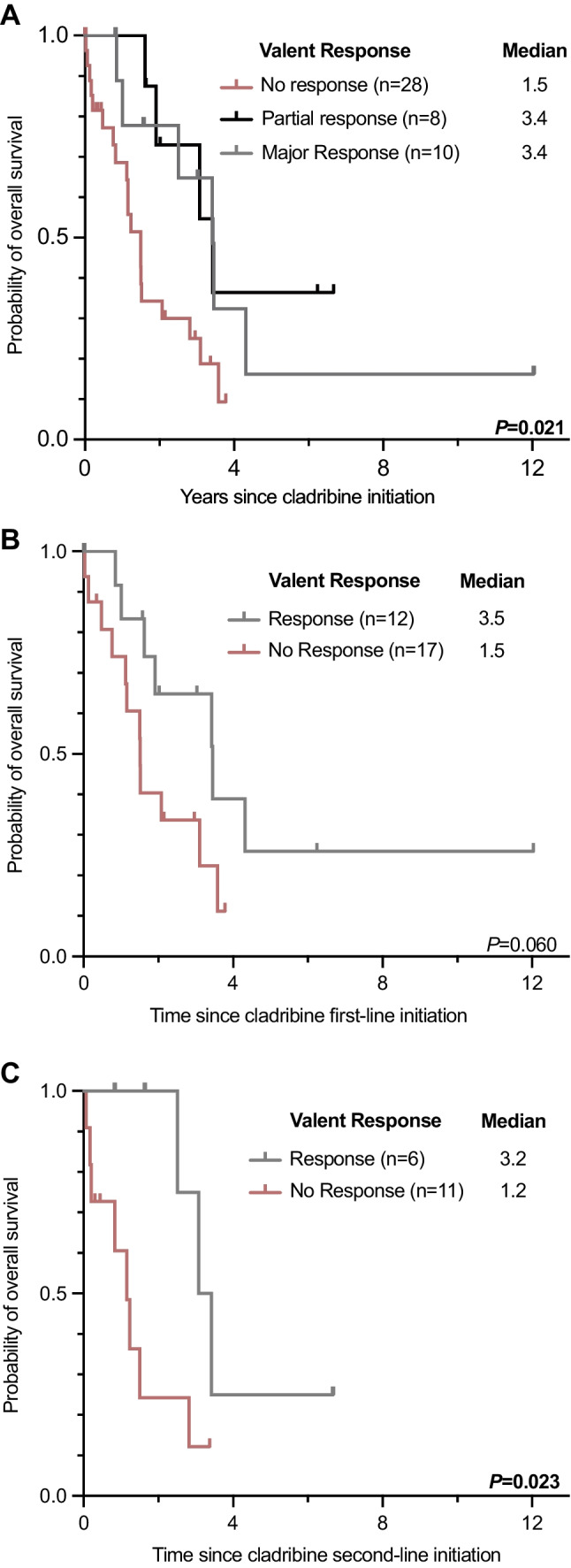
Kaplan-Meier estimates of overall survival according to **(A)** the first- and second-line use of cladribine, **(B)** the Mutation-Adjusted Risk Score (MARS) and **(C)** the International Prognostic Scoring System for Advanced Systemic Mastocytosis (IPSM-AdvSM)

According to modified Valent criteria, the overall response rate (ORR) on cladribine in 46/79 (58%) evaluable patients was 18/46 (39%) with a complete remission (CR) in 0/46, a major remission (MR) in 10/46 (22%), and a partial remission (PR) in 8/46 (17%) patients. Comparisons between the patient cohorts with and without available response assessment revealed balanced subgroups (Appendix Table 1). There was no difference between 1L- (12/29, 41%) and 2L-treatment (6/17, 35%; *P* = 0.690). Any response (MR + PR) vs. no response was associated with improved median OS (3.4 vs. 1.5 years, *P* = 0.021; Fig. 1A) and was independent of 1L- (3.5 vs. 1.5 years, *P* = 0.060) or 2L- (3.2 vs. 1.2 years, *P* = 0.023) treatment (Figs. 1B-C). The use of ≥ 3 cycles was associated with an improved ORR (14/25, 56% vs. 4/21, 19% responder; *P* = 0.011) and median OS (2.8 vs. 1.2 years, *P* = 0.038). The median OS (1.9 vs. 1.2 years, *P* = 0.311) was not different between 1L- and 2L-treatment (Fig. 2A, Table 1).

**Fig. 3:**
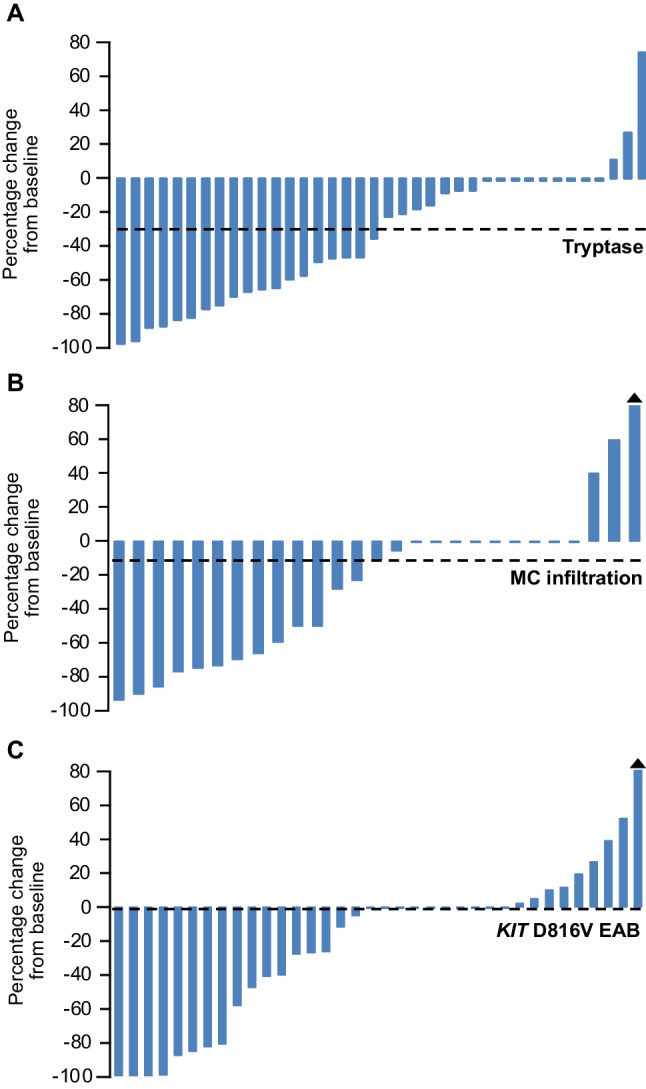
(**A**) Best percentage change of (A) serum tryptase, (B) bone marrow mast cell infiltration and (C) *KIT* D816V expressed allele burden. The dashed line displays the median change. The triangle indicates percentage change >60%

The median percentage change from baseline to response assessment of serum tryptase, BM MC infiltration and *KIT* D816V EAB was -29% (range -97% to 75%), 11% (range -94% to 233%) and -1% (range -100% to 1669%; Fig. 3), respectively. The median percentage change was significantly higher in responders vs. non-responders according to modified Valent criteria (serum tryptase -46% vs. -28%, BM MC infiltration -50% vs. 0% and *KIT* D816V EAB -41% vs. 0%; *P* < 0.05).

### Risk stratification according to recently established prognostic scoring systems

MARS [7] and the IPSM-AdvSM [34] were recently validated for up-front midostaurin risk-stratification [23]. Both risk scores were assessed for stratification at time of diagnosis (all patients) and at time of initiation of 1L- or 2L-treatment. At diagnosis, median OS according to MARS (*n* = 69 evaluable) was 1.5, 2.1, and 1.9 years in low- (*n* = 16, 23%), intermediate- (*n* = 11, 16%) and high-risk patients (*n* = 42, 61%, *P* = 0.270), respectively. Median OS according to IPSM-AdvSM (*n* = 71 evaluable) was 1.3, 2.5, and 1.2 years in AdvSM-1/2 (*n* = 16, 23%), AdvSM-3 (*n* = 36, 50%), and AdvSM-4 patients (*n* = 19, 27%, *P* = 0.053; Fig. 2B-C), respectively. Data were not different when applied at start of 1L- (*P* = 0.592, *P* = 0.769) or 2L-treatment (*P* = 0.125, *P* = 0.054). Of note, neither MAPS (*P* = 0.358) nor GPSM (*P* = 0.127) were able to predict OS on cladribine (Appendix Figure 1).

### Univariate and multivariable analyses

**Fig. 4:**
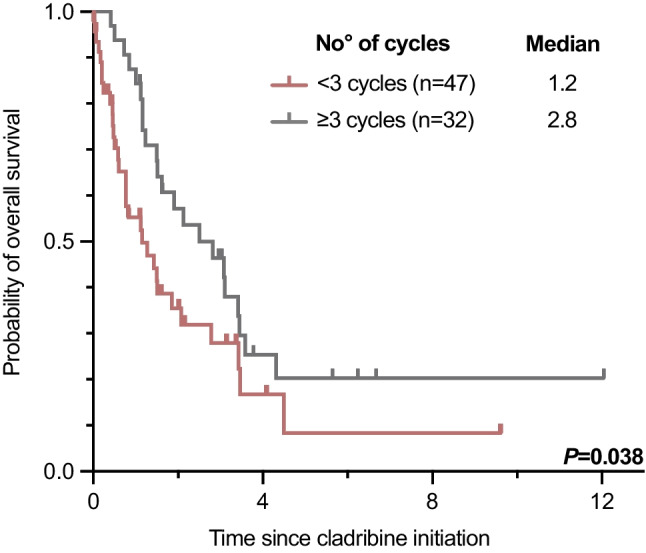
Kaplan–Meier estimates of overall survival in cladribine treated patients with ≥ / < 3 cycles

**Fig. 5:**
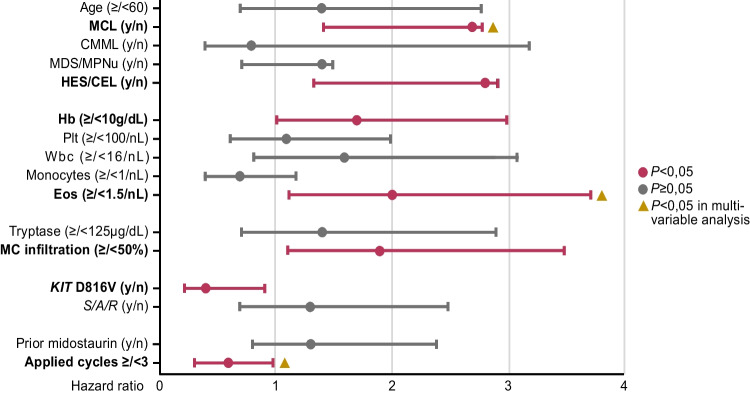
Univariate and multivariable analysis of baseline parameters (entire cohort). Abbreviations: Eos, eosinophils; CMML chronic myelomonocytic leukemia; Hb, hemoglobin; HES/CEL, hypereosinophilic syndrome/chronic eosinophilic leukemia; MC, mast cell; MCL, mast cell leukemia; MDS/MPNu, myelodysplastic/myeloproliferative neoplasms unclassifiable; Plt, platelets; *S/A/R, SRSF2/ASXL1/RUNX1*; Wbc, white blood cells

**Fig. 6:**
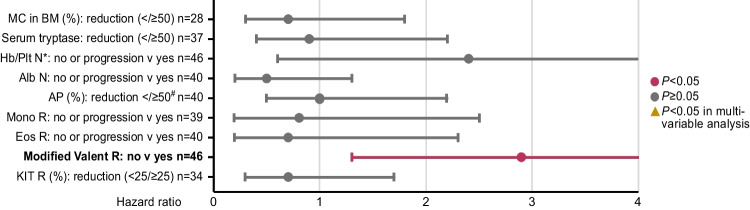
Univariate analysis of on-treatment parameters. *Cheson criteria for transfusion were considered if necessary. ^#^or normalization. Abbreviations: AP, alkaline phosphatase; BM, Bone marrow; CI, confidence interval; Eos, eosinophilia; Hb, hemoglobin; MC, mast cell; Mono, monocytosis; N, normalization; HR, Hazard ratio; MC, mast cell; Plt, platelets; R, response

Univariate and multivariable analyses of baseline parameters from all 79 patients identified diagnosis of MCL (hazard ratio [HR] 3.5, 95% confidence interval [CI, 1.3–9.1], *P* = 0.012), eosinophilia ≥ 1.5 × 10^9^/L (HR 2.9 [CI 1.4–6.2], *P* = 0.006) and application of < 3 cycles cladribine (HR 0.4 [CI 0.2–0.8], *P* = 0.008) as independent adverse prognostic parameters for OS (Figs. 4 and 5, Appendix Figure 2, Appendix Table 3). Outcome on cladribine was independent of the presence of one or more additional somatic mutations in the *S/A/R* gene panel (HR 0.6 [CI 0.2–2.0], *P* = 0.412). In univariate analysis, modified Valent criteria were superior (HR 2.9 [CI 1.3–6.6], *P* = 0.026; Fig. 6; Appendix Table 4) to a single factor-based on-treatment response assessment, e.g. BM MC infiltration, serum tryptase or *KIT* D816V EAB.

## Discussion

In historical cohorts of up to a maximum of 32 AdvSM patients [18, 19, 21], the ORR on cladribine according to (modified) Valent criteria [21, 36] ranged between 50 and 100%. [20] Further interpretation on the impact of treatment with cladribine on progression-free (PFS), relapse-free (RFS), event-free (EFS), leukemia-free (LFS) and overall survival is limited because (i) most reports did not clearly differentiate between ISM and AdvSM, (ii) no report separated between 1L- and 2L-treatment and (iii) the definitions of PFS/RFS/EFS/LFS were not consistent between studies. In a registry-based cross-assessment, we recently reported an ORR (modified Valent criteria) of 35% in midostaurin-treated and 40% in cladribine-treated patients [23]. Notwithstanding, the OS on cladribine was significantly inferior to midostaurin in both 1L- and 2L-treatment cohorts. In the current report, we sought to provide a more detailed analysis on response rates on cladribine in 1L- and 2L-treatment, biomarkers indicating response and resistance and the association between ORR and OS.

Multivariable analysis identified hypereosinophilia (> 1.5 × 10^9^/l), as marker of an AHN, diagnosis of MCL, and application < 3 cycles as adverse prognostic markers. This confirms a recent report from the Mayo Clinic registry on 22 cladribine-treated AdvSM patients indicating a diagnosis of an AHN (in addition to older age and absence of *KIT* D816V) as adverse prognostic markers for survival and is also in line with a previous publication on the poor prognostic impact of eosinophilia in SM [18, 37]. Recent data also revealed that midostaurin was superior to cladribine in controlling AHN-associated myeloproliferation [23]. The application of ≥ 3 cycles was further associated with a higher ORR.

In a minority of patients (< 10%), cladribine was used for bridging the interval to the start of the midostaurin trial in 2009 and at later time points, it was used in a few patients for more rapid MC debulking with subsequent pre-planned switch to midostaurin. Although myelosuppression became apparent in approximately 20% of patients, infectious complications were not noted as reasons for treatment discontinuation. In contrast to midostaurin, OS on cladribine was not influenced by cytopenias prior to treatment or additional somatic mutations in the *S*/*A*/*R* gene panel. Consequently, none of the prognostic scoring systems (MARS, IPSM, MAPS, GPSM) was predictive for OS. The reasons for this observation are unknown but may be explained at least in part by the fact that the scores more effectively identify low-risk patients on targeted treatment with midostaurin [23, 27] or avapritinb [26] than on conventional chemotherapy with cladribine.

In contrast to the recent report from the Mayo Clinic, possibly due to the higher number of patients in our study, any response according to modified Valent criteria in 1L- but also 2L-treatment was associated with improved OS, thus confirming the usefulness of response assessment for guiding further treatment strategies. The data were underscored by the predictive superiority of modified Valent criteria versus a single factor-based response assessment. Although 2L patients presented with a higher disease burden, response and survival were not statistically different from 1L patients.

Recently reported propensity score weighted analyses on LFS/EFS and OS revealed superiority of midostaurin over cladribine and of avapritinib over best available treatment including midostaurin and cladribine [23–25]. However, we conclude that (i) cladribine remains a relevant option within the AdvSM treatment algorithm; its application in 1L-, 2L- or 3L-line locally depends on the approval status and availability of midostaurin and avapritinib; (ii) mast cell leukemia, eosinophilia, application of < 3 cycles and lack of response according to modified Valent criteria are adverse prognostic markers, and (iii) commonly used prognostic models for AdvSM are of limited value because of high mortality in low- and intermediate-risk patients.

The genetic and clinical complexity of AdvSM requires further prospective clinical trials to study the effects of KIT inhibitors in combination with simultaneous or intermittent use of other anti-neoplastic drugs, e.g. cladribine or hypomethylating agents. Such an approach may counteract the potential outgrowth of *KIT* D816V negative or multimutated subclones [38]. For patients with progression into secondary MCL or secondary AML, AML-like chemotherapy with or without subsequent allogeneic stem cell transplantation remains the most reasonable and potentially curative treatment options.

## Supplementary Information

Below is the link to the electronic supplementary material.

Supplementary file1 (PDF 178 KB)

## Data Availability

The data sets used and/or analyzed during the current study are available from the corresponding author (A.R.) on reasonable request.

## References

[1] Valent P, Akin C, Metcalfe DD (2017). Mastocytosis: 2016 updated WHO classification and novel emerging treatment concepts. Blood.

[2] Valent P, Akin C, Escribano L (2007). Standards and standardization in mastocytosis: consensus statements on diagnostics, treatment recommendations and response criteria. Eur J Clin Invest.

[3] Valent P, Akin C, Sperr WR (2003). Diagnosis and treatment of systemic mastocytosis: state of the art. Br J Haematol.

[4] Pardanani A (2021). Systemic mastocytosis in adults: 2021 Update on diagnosis, risk stratification and management. Am J Hematol.

[5] Chatterjee A, Ghosh J, Kapur R (2015). Mastocytosis: a mutated KIT receptor induced myeloproliferative disorder. Oncotarget.

[6] Jawhar M, Schwaab J, Schnittger S (2015). Molecular profiling of myeloid progenitor cells in multi-mutated advanced systemic mastocytosis identifies KIT D816V as a distinct and late event. Leukemia.

[7] Jawhar M, Schwaab J, Alvarez-Twose I (2019). MARS: mutation-adjusted risk score for advanced systemic mastocytosis. J Clin Oncol.

[8] Jawhar M, Schwaab J, Schnittger S (2016). Additional mutations in SRSF2, ASXL1 and/or RUNX1 identify a high-risk group of patients with KIT D816V(+) advanced systemic mastocytosis. Leukemia.

[9] Jawhar M, Schwaab J, Hausmann D (2016). Splenomegaly, elevated alkaline phosphatase and mutations in the SRSF2/ASXL1/RUNX1 gene panel are strong adverse prognostic markers in patients with systemic mastocytosis. Leukemia.

[10] Schwaab J, Schnittger S, Sotlar K (2013). Comprehensive mutational profiling in advanced systemic mastocytosis. Blood.

[11] Muñoz-González JI, Álvarez-Twose I, Jara-Acevedo M (2021). Proposed global prognostic score for systemic mastocytosis: a retrospective prognostic modelling study. Lancet Haematol.

[12] Pardanani A, Shah S, Mannelli F (2018). Mayo alliance prognostic system for mastocytosis: clinical and hybrid clinical-molecular models. Blood Adv.

[13] DeAngelo DJ, George TI, Linder A (2018). Efficacy and safety of midostaurin in patients with advanced systemic mastocytosis: 10-year median follow-up of a phase II trial. Leukemia.

[14] Gotlib J, Kluin-Nelemans HC, George TI (2016). Efficacy and safety of midostaurin in advanced systemic mastocytosis. N Engl J Med.

[15] Chandesris MO, Damaj G, Canioni D (2016). Midostaurin in advanced systemic mastocytosis. N Engl J Med.

[16] DeAngelo DJ, Radia DH, George TI (2021). Safety and efficacy of avapritinib in advanced systemic mastocytosis: the phase 1 EXPLORER trial. Nat Med.

[17] Gotlib J, Reiter A, Radia DH (2021). Efficacy and safety of avapritinib in advanced systemic mastocytosis: interim analysis of the phase 2 PATHFINDER trial. Nat Med.

[18] Tefferi A, Kittur J, Farrukh F (2021). Cladribine therapy for advanced and indolent systemic mastocytosis: Mayo Clinic experience in 42 consecutive cases. Br J Haematol.

[19] Barete S, Lortholary O, Damaj G (2015). Long-term efficacy and safety of cladribine (2-CdA) in adult patients with mastocytosis. Blood.

[20] Kluin-Nelemans HC, Oldhoff JM, Van Doormaal JJ (2003). Cladribine therapy for systemic mastocytosis. Blood.

[21] Lim KH, Pardanani A, Butterfield JH, Li CY, Tefferi A (2009). Cytoreductive therapy in 108 adults with systemic mastocytosis: Outcome analysis and response prediction during treatment with interferon-alpha, hydroxyurea, imatinib mesylate or 2-chlorodeoxyadenosine. Am J Hematol.

[22] Jawhar M, Schwaab J, Meggendorfer M (2017). The clinical and molecular diversity of mast cell leukemia with or without associated hematologic neoplasm. Haematologica.

[23] Lübke J, Schwaab J, Naumann N (2022). Superior efficacy of midostaurin over cladribine in advanced systemic mastocytosis: a registry-based analysis. J Clin Oncol.

[24] Pilkington H, Smith S, Roskell N, Iannazzo S (2022). Indirect treatment comparisons of avapritinib versus midostaurin for patients with advanced systemic mastocytosis. Future Oncol.

[25] Reiter A, Gotlib J, Álvarez-Twose I (2022). Efficacy of avapritinib versus best available therapy in the treatment of advanced systemic mastocytosis. Leukemia.

[26] Reiter A, Schwaab J, DeAngelo DJ (2022). Efficacy and safety of avapritinib in previously treated patients with advanced systemic mastocytosis. Blood Adv.

[27] Jawhar M, Schwaab J, Naumann N (2017). Response and progression on midostaurin in advanced systemic mastocytosis: KIT D816V and other molecular markers. Blood.

[28] Lübke J, Naumann N, Kluger S (2019). Inhibitory effects of midostaurin and avapritinib on myeloid progenitors derived from patients with KIT D816V positive advanced systemic mastocytosis. Leukemia.

[29] Valent P, Horny HP, Escribano L (2001). Diagnostic criteria and classification of mastocytosis: a consensus proposal. Leuk Res.

[30] Reiter A, George TI, Gotlib J (2020). New developments in diagnosis, prognostication, and treatment of advanced systemic mastocytosis. Blood.

[31] Khoury JD, Solary  E, Abla O (2022). he 5th edition of the World Health Organization classification of haematolymphoid tumours: myeloid and histiocytic/dendritic neoplasms. Leukemia.

[32] Jawhar M, Schwaab J, Horny HP (2016). Impact of centralized evaluation of bone marrow histology in systemic mastocytosis. Eur J Clin Invest.

[33] Erben P, Schwaab J, Metzgeroth G (2014). The KIT D816V expressed allele burden for diagnosis and disease monitoring of systemic mastocytosis. Ann Hematol.

[34] Sperr WR, Kundi M, Alvarez-Twose I (2019). International prognostic scoring system for mastocytosis (IPSM): a retrospective cohort study. Lancet Haematol.

[35] Naumann N, Lübke J, Baumann S (2021). Adverse prognostic impact of the KIT D816V transcriptional activity in advanced systemic mastocytosis. Int J Mol Sci.

[36] Valent P, Akin C, Sperr WR (2003). Aggressive systemic mastocytosis and related mast cell disorders: current treatment options and proposed response criteria. Leuk Res.

[37] Kluin-Nelemans HC, Reiter A, Illerhaus A (2019). Prognostic impact of eosinophils in mastocytosis: analysis of 2350 patients collected in the ECNM Registry. Leukemia.

[38] Jawhar M, Dohner K, Kreil S (2019). KIT D816 mutated/CBF-negative acute myeloid leukemia: a poor-risk subtype associated with systemic mastocytosis. Leukemia.

