# Supplementary material for: Response and resistance to cladribine in patients with advanced systemic mastocytosis: a registry-based analysis
Source: Ann Hematol. 2023 Apr 4;102(8):2077–85. doi: 10.1007/s00277-023-05180-y (PMC10344834; doi:10.1007/s00277-023-05180-y)
Supplement: Supplementary file 1 — Supplementary file1 (PDF 178 KB) [file 277_2023_5180_MOESM1_ESM.pdf]

Appendix Figure 1. Kaplan-Meier estimates of overall survival according to (A) the Mayo Alliance Prognostic System (MAPS) and (B) the Global Prognostic Score for Systemic Mastocytosis (GPSM).

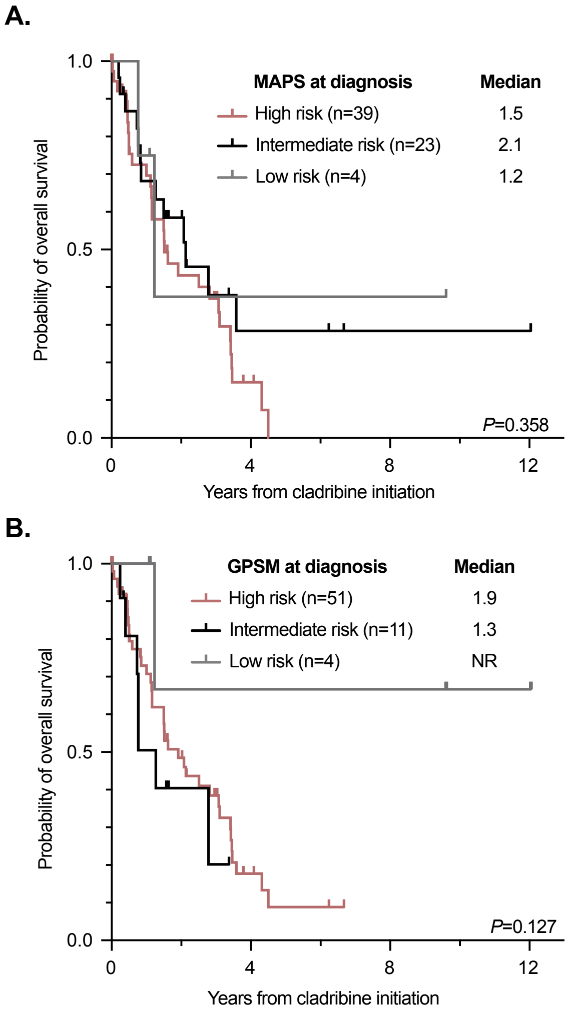

Appendix Figure 2. Univariate and multivariable analysis of baseline parameters from a modified cohort after exclusion of patients with prior treatment (n=31) and censoring patients at start of subsequent treatment, as potentially confounding treatment-associated parameters, revealed leukocytosis ≥16 x 10^9/L (HR 5.0, 95% confidence interval [CI 1.2-21.0], *P*=0.026) and eosinophilia ≥1.5 x 10^9/L (HR 3.0 [CI 1.0-8.8], *P*=0.048) as adverse prognostic markers for OS. Abbreviations: Eos, eosinophils; CMML chronic myelomonocytic leukemia; Hb, hemoglobin; HES/CEL, hypereosinophilic syndrome/chronic eosinophilic leukemia; MC, mast cell; MCL, mast cell leukemia; MDS/MPNu, myelodysplastic/myeloproliferative neoplasms unclassifiable; Plt, platelets; *S/A/R, SRSF2/ASXL1/RUNX1*; Wbc, white blood cells.

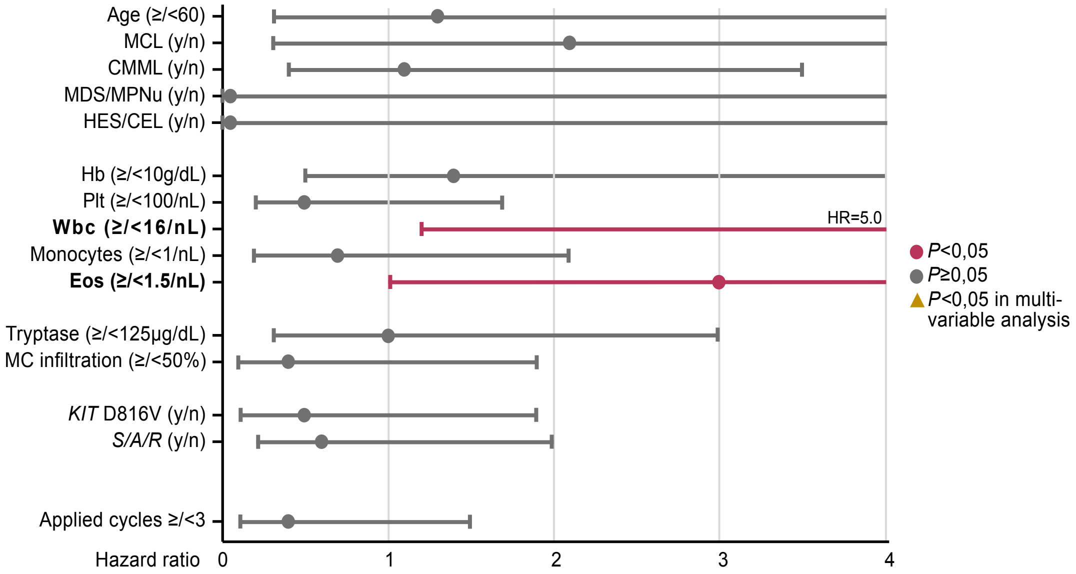

Appendix Table 1: Demographic and disease characteristics cladribine treated patients stratified according to availability for response assessment

	Response assessment available	Response assessment not available	*P*
Number of patients at baseline, *n* (%)	46 (58)	33 (42)	
Age in years at treatment initiation; median (range)	69 (27-87)	67 (45-84)	0.207
Male, *n* (%)	33 (72)	20 (61)	0.299
**Diagnosis**			
ASM, *n* (%)	3 (7)	6 (18)	0.108
SM-AHN, *n* (%)	35 (76)	21 (64)	0.230
MCL±AHN, *n* (%)	8 (17)	6 (18)	0.928
**C-findings**			
Hemoglobin, g/dL; median (range)	11 (7-15)	10 (7-13)	0.496
<10g/dL, *n* (%)	18 (40)	12 (50)	0.425
Platelets, x10^9/L; median (range)	99 (12-312)	97 (25-630)	0.263
<100x10^9/L, *n* (%)	23 (51)	13 (54)	0.809
ANC, x10^9/L; median (range)	5 (0-65)	6 (2-62)	0.453
<1x10^9/L, *n* (%)	2 (4)	0 (0)	0.315
Alkaline phosphatase, U/L; median (range)	300 (67-1464)	180 (45-1736)	0.485
>150U/L, *n* (%)	40 (89)	16 (67)	0.025
Albumin level, g/L; median (range)	33 (15-44)	36 (25-48)	0.047
<34g/L, *n* (%)	25 (56)	5 (29)	0.066
Weight loss (>10 % over last 6 months), *n* (%)	33 (83)	12 (80)	0.831
**Other relevant findings**			
Leukocytes, x10^9/L; median (range)	9.1 (2.6-104.4)	10.8 (1.3-142.2)	0.222
Monocytes, x10^9/L; median (range)	1.0 (0.0-5.2)	0.9 (0.0-18.5)	0.133
>1x10^9/L, *n* (%)	21 (48)	10 (44)	0.741
Eosinophils, x10^9/L; median (range)	0.4 (0.0-9.3)	0.8 (0.0-68.3)	0.152
>1.5x10^9/L, *n* (%)	12 (27)	9 (39)	0.293
MC-infiltration in BM biopsy, %; median (range)	50 (5-100)	45 (3-90)	0.474
Serum tryptase level, µg/L; median (range)	221 (24-1200)	200 (23-1150)	0.406
Serum tryptase level, >100µg/L, *n* (%)	40 (89)	15 (71)	0.076
Serum tryptase level, >200µg/L, *n* (%)	24 (53)	12 (57)	0.772
Serum tryptase level, >400µg/L, *n* (%)	17 (38)	5 (24)	0.262
Splenomegaly, *n* (%)	42 (94)	22 (96)	0.700
Hepatomegaly, *n* (%)	29 (69)	14 (67)	0.848
Lymphadenopathy, *n* (%)	35 (80)	14 (67)	0.260
KIT D816V EAB in PB, %, median (range)	35 (0-80)	37 (0-55)	0.811
**MARS score at diagnosis**, *n* (%)			
Low-risk, *n* (%)	7 (16)	8 (33)	0.089
Intermediate-risk, *n* (%)	11 (24)	6 (25)	0.959
High-risk, *n* (%)	27 (60)	10 (42)	0.146
**Treatment and outcome**			
Follow-up, years since diagnosis; median (range)	2.6 (0.1-17.0)	2.3 (0.2-16.5)	0.500
Follow-up, years since 1st cycle; median (range)	1.6(0.0-12.0)	1.1 (0.0-9.6)	0.306
Years to treatment since diagnosis; median (range)	0.7 (0.0-11.0)	0.8 (0.1-6.8)	0.948
Years of treatment duration; median (range)	0.3 (0.0-2.4)	0.3 (0.0-0.8)	0.154
Number of cladribine cycles, median (range)	4 (1-8)	2 (1-5)	<0.001
Cycles per months, median (range)	0.99 (0.4-4.8)	1.1 (0.7-3.3)	0.326
Deaths, *n* (%)	30 (65)	23 (70)	0.676
Median OS, years (95% CI)	2.1 (0.7-3.5)	1.2 (0.4-1.9)	0.249

ANC, absolute neutrophil count; ASM, aggressive systemic mastocytosis; BM, bone marrow; CI, confidence interval; EAB, expressed allele burden; MARS, mutation-adjusted risk score; MC, mast cell; MCL±AHN, mast cell leukemia with/without an associated hematologic neoplasm; NR, monocytosis non-response; OS, overall survival; PB, peripheral blood; R, monocytosis response; SM-AHN, systemic mastocytosis with an associated hematological neoplasm.

Appendix Table 2: Demographic and disease characteristics of 79 cladribine treated stratified according first- and second-line.

	All	First-line	Second-line	*P*
Number of patients at baseline, *n* (%)	79	48 (61)	31 (39)	
Age in years at treatment initiation; median (range)	68 (27-87)	69 (27-81)	66 (48-87)	0.770
Male, *n* (%)	53 (79)	32 (48)	21 (68)	0.921
**Diagnosis**				
ASM, *n* (%)	9 (11)	7 (15)	2 (7)	0.267
SM-AHN, *n* (%)	56 (71)	35 (73)	21 (68)	0.621
MCL±AHN, *n* (%)	14 (18)	6 (13)	8 (26)	0.130
**C-findings**				
Hemoglobin, g/dL; median (range)	10 (7-15)	11 (7-13)	9 (7-15)	0.124
<10g/dL, *n* (%)	30 (44)	16 (35)	14 (61)	0.039
Platelets, $x10^9/L$; median (range)	99 (12-630)	105 (12-630)	87 (25-388)	0.254
$<100x10^9/L$, *n* (%)	36 (52)	23 (50)	13 (57)	0.609
ANC, $x10^9/L$; median (range)	5 (0-65)	6 (1-65)	4 (0-62)	0.648
$<1x10^9/L$, *n* (%)	2 (3)	1 (2)	1 (4)	0.636
Alkaline phosphatase, U/L; median (range)	270 (45-1736)	242 (45-1736)	300 (63-919)	0.580
>150U/L, *n* (%)	56 (81)	37 (80)	19 (83)	0.828
Albumin level, g/L; median (range)	34 (15-48)	34 (21-44)	34 (15-48)	0.709
<34g/L, *n* (%)	30 (48)	19 (49)	11 (48)	0.946
Weight loss (>10 % over last 6 months), *n* (%)	45 (82)	28 (80)	17 (85)	0.644
**Other relevant findings**				
Leukocytes, $x10^9/L$; median (range)	9.8 (1.3-14.2)	10.4 (1.3-10.4)	9.0 (2.6-14.2)	0.799
Monocytes, $x10^9/L$; median (range)	0.9 (0.0-18.5)	1.1 (0.0-17.9)	0.9 (0-18.5)	0.862
$>1x10^9/L$, *n* (%)	31 (46)	23 (52)	8 (35)	0.173
Eosinophils, $x10^9/L$; median (range)	0.5 (0.0-68.3)	0.5 (0.0-1.4)	0.3 (0.0-68.3)	0.254
$>1.5x10^9/L$, *n* (%)	21 (31)	14 (31)	7 (30)	0.955
MC-infiltration in BM biopsy, %; median (range)	45 (3-100)	40 (5-100)	58 (3-90)	0.023
Serum tryptase level, µg/L; median (range)	215 (23-1200)	199 (23-1150)	448 (54-1200)	0.018
Serum tryptase level, >100µg/L, *n* (%)	55 (83)	36 (82)	19 (86)	0.640
Splenomegaly, *n* (%)	64 (94)	41 (91)	23 (100)	0.141
Hepatomegaly, *n* (%)	43 (68)	27 (68)	16 (70)	0.865
Lymphadenopathy, *n* (%)	49 (75)	33 (77)	19 (73)	0.722
KIT D816V EAB in PB, %, median (range)	35 (0-80)	35 (0-61)	37 (0-80)	0.409
**MARS score at diagnosis**, *n* (%)	69 (87)	40 (83)	29 (94)	
Low-risk, *n* (%)	16 (23)	10 (25)	6 (21)	0.675
Intermediate-risk, *n* (%)	11 (16)	6 (15)	5 (17)	0.802
High-risk, *n* (%)	42 (61)	24 (60)	18 (62)	0.862
**Treatment and outcome**				
Follow-up, years since diagnosis; median (range)	2.5 (0.1-17.0)	2.6 (0.1-17.0)	2.2 (0.1-16.4)	0.821
Follow-up, years since 1st cycle; median (range)	1.2 (0.0-12.0)	1.5 (0.0-12-0)	0.8 (0.0-9.6)	0.186
Years to treatment since diagnosis; median (range)	0.7 (0.0-11.0)	0.5 (0.0-10.1)	1.0 (0.1-8.8)	0.083
Years of treatment duration; median (range)	0.3 (0.0-2.4)	0.3 (0.0-1.3)	0.3 (0.0-2.4)	0.612
Number of cladribine cycles, median (range)	3 (1-8)	3 (1-6)	3 (1-8)	0.743
Cycles per months, median (range)	1.0 (0.4-4.8)	1.0 (0.4-4.0)	1.0 (0.7-4.8)	0.848
Deaths, *n* (%)	53 (67)	34 (71)	19 (61)	0.378
Median OS, years (95% CI)	1.5 (1.0-2.0)	1.9 (1.1-2.6)	1.2 (0.3-2.1)	0.311

ANC, absolute neutrophil count; ASM, aggressive systemic mastocytosis; BM, bone marrow; CI, confidence interval; EAB, expressed allele burden; MARS, mutation-adjusted risk score; MC, mast cell; MCL±AHN, mast cell leukemia with/without an associated hematologic neoplasm; NR, monocytosis non-response; OS, overall survival; PB, peripheral blood; R, monocytosis response; SM-AHN, systemic mastocytosis with an associated hematological neoplasm.

Appendix Table 3: Demographic and disease characteristics of 79 cladribine treated stratified according to applied cycles

	All	≥3 cycles	<3 cycles	*P*
Number of patients at baseline, *n* (%)	79	32 (41)	47 (59)	
Age in years at treatment initiation; median (range)	68 (27-87)	69 (45-81)	68 (27-87)	0.979
Male, *n* (%)	53 (79)	22 (69)	31 (66)	0.795
**Diagnosis**				
ASM, *n* (%)	9 (11)	3 (9)	6 (13)	0.641
SM-AHN, *n* (%)	56 (71)	25 (78)	31 (66)	0.243
MCL±AHN, *n* (%)	14 (18)	4 (13)	10 (21)	0.316
**C-findings**				
Hemoglobin, g/dL; median (range)	10 (7-15)	11 (8-15)	10 (7-13)	0.273
<10g/dL, *n* (%)	30 (44)	12 (40)	18 (46)	0.609
Platelets, x10^9/L; median (range)	99 (12-630)	105 (26-312)	96 (12-630)	0.432
<100x10^9/L, *n* (%)	36 (52)	15 (50)	21 (54)	0.751
ANC, x10^9/L; median (range)	5 (0-65)	5 (1-65)	6 (0-62)	0.928
<1x10^9/L, *n* (%)	2 (3)	1 (3)	1 (3)	0.880
Alkaline phosphatase, U/L; median (range)	270 (45-1736)	328 (82-1464)	205 (45-1736)	0.098
>150U/L, *n* (%)	56 (81)	26 (87)	30 (77)	0.305
Albumin level, g/L; median (range)	34 (15-48)	35 (15-44)	33 (21-48)	0.666
<34g/L, *n* (%)	30 (48)	12 (41)	18 (55)	0.301
Weight loss (>10 % over last 6 months), *n* (%)	45 (82)	21 (78)	24 (86)	0.446
**Other relevant findings**				
Leukocytes, x10^9/L; median (range)	9.8 (1.3-14.2)	9.4 (2.6-10.4)	10.0 (1.3-4.2)	0.902
Monocytes, x10^9/L; median (range)	0.9 (0.0-18.5)	1.1 (0.0-7.1)	0.7 (0.0-18.5)	0.454
>1x10^9/L, *n* (%)	31 (46)	17 (57)	14 (38)	0.124
Eosinophils, x10^9/L; median (range)	0.5 (0.0-68.3)	0.5 (0.0-35.1)	0.4 (0.0-68.3)	0.725
>1.5x10^9/L, *n* (%)	21 (31)	6 (20)	11 (29)	0.398
MC-infiltration in BM biopsy, %; median (range)	45 (3-100)	50 (3-90)	40 (3-90)	0.276
Serum tryptase level, µg/L; median (range)	215 (23-1200)	271 (43-1200)	188 (23-1118)	0.486
Serum tryptase level, >100µg/L, *n* (%)	55 (83)	28 (93)	27 (75)	0.047
Serum tryptase level, >200µg/L, *n* (%)	36 (55)	20 (67)	16 (44)	0.071
Serum tryptase level, >400µg/L, *n* (%)	21 (34)	11 (37)	10 (32)	0.717
Splenomegaly, *n* (%)	64 (94)	28 (93)	36 (95)	0.807
Hepatomegaly, *n* (%)	43 (68)	20 (69)	19 (66)	0.780
Lymphadenopathy, *n* (%)	49 (75)	24 (83)	21 (68)	0.180
KIT D816V EAB in PB, %, median (range)	35 (0-80)	37 (0-80)	35 (0-72)	0.370
**MARS score at diagnosis**, *n* (%)	69 (87)	30 (43)	39 (57)	
Low-risk, *n* (%)	16 (23)	4 (13)	12 (31)	0.089
Intermediate-risk, *n* (%)	11 (16)	5 (17)	6 (15)	0.885
High-risk, *n* (%)	42 (61)	21 (70)	21 (54)	0.173
**Treatment and outcome**				
Follow-up, years since diagnosis; median (range)	2.5 (0.1-17.0)	3.4 (0.5-17.0)	1.9 (0.1-16.5)	0.270
Follow-up, years since 1st cycle; median (range)	1.2 (0.0-12.0)	2.0 (0.4-12.0)	0.8 (0.0-9.6)	0.007
Years to treatment since diagnosis; median (range)	0.7 (0.0-11.0)	0.7 (0.0-5.0)	0.8 (0.0-11.0)	0.221
Years of treatment duration; median (range)	0.3 (0.0-2.4)	0.4 (0.3-2.4)	0.2 (0.0-0.8)	<0.001
Number of cladribine cycles, median (range)	3 (1-8)	5 (4-8)	2 (1-3)	<0.001
Cycles per months, median (range)	1.0 (0.4-4.8)	1.0 (0.8-4.8)	1.0 (0.4-3.3)	0.242
Deaths, *n* (%)	53 (67)	22 (69)	31 (66)	0.795
Median OS, years (95% CI)	1.5 (1.0-2.0)	2.8 (1.4-4.2)	1.2 (0.3-2.0)	0.038

ANC, absolute neutrophil count; ASM, aggressive systemic mastocytosis; BM, bone marrow; CI, confidence interval; EAB, expressed allele burden; MARS, mutation-adjusted risk score; MC, mast cell; MCL±AHN, mast cell leukemia with/without an associated hematologic neoplasm; NR, monocytosis non-response; OS, overall survival; PB, peripheral blood; R, monocytosis response; SM-AHN, systemic mastocytosis with an associated hematological neoplasm.

Table 4: Demographic and disease characteristics of 46 cladribine treated stratified according to response status

	Responder	Non-Responder	*P*
Number of patients at baseline, *n* (%)	18 (39)	28 (61)	
Age in years at treatment initiation; median (range)	68 (49-77)	69 (27-87)	0.404
Male, *n* (%)	12 (67)	21 (75)	0.540
**Diagnosis**			
ASM, *n* (%)	3 (17)	0 (0)	0.026
SM-AHN, *n* (%)	14 (78)	21 (75)	0.829
MCL±AHN, *n* (%)	1 (6)	7 (25)	0.090
**C-findings**			
Hemoglobin, g/dL; median (range)	11 (8-12)	10 (7-15)	0.747
<10g/dL, *n* (%)	7 (41)	11 (39)	0.900
Platelets, $x10^9$/L; median (range)	114 (37-312)	82 (12-297)	0.274
<$100x10^9$/L, *n* (%)	8 (47)	15 (54)	0.672
ANC, $x10^9$/L; median (range)	6 (1-28)	5 (0-65)	0.490
<$1x10^9$/L, *n* (%)	0 (0)	2 (7)	0.260
Alkaline phosphatase, U/L; median (range)	261 (67-1028)	346 (117-1464)	0.571
>150U/L, *n* (%)	14 (82)	26 (93)	0.277
Albumin level, g/L; median (range)	33 (22-44)	32 (15-42)	0.631
<34g/L, *n* (%)	10 (59)	15 (54)	0.731
Weight loss (>10 % over last 6 months), *n* (%)	14 (88)	19 (79)	0.497
**Other relevant findings**			
Leukocytes, $x10^9$/L; median (range)	9.6 (2.6-39.3)	9.0 (2.6-104.4)	0.332
Monocytes, $x10^9$/L; median (range)	1.0 (0.0-3.7)	0.9 (0.1-5.2)	0.208
>$1x10^9$/L, *n* (%)	9 (53)	12 (44)	0.583
Eosinophils, $x10^9$/L; median (range)	0.3 (0.0-2.1)	0.4 (0.0-9.2)	0.081
>$1.5x10^9$/L, *n* (%)	4 (24)	8 (29)	0.711
MC-infiltration in BM biopsy, %; median (range)	50 (10-100)	50 (5-90)	0.535
Serum tryptase level, µg/L; median (range)	220 (24-1200)	246 (54-1118)	0.858
Serum tryptase level, >100µg/L, *n* (%)	15 (88)	25 (89)	0.913
Serum tryptase level, >200µg/L, *n* (%)	9 (53)	15 (54)	0.967
Serum tryptase level, >400µg/L, *n* (%)	6 (35)	11 (39)	0.789
Splenomegaly, *n* (%)	16 (94)	26 (93)	0.870
Hepatomegaly, *n* (%)	11 (65)	18 (72)	0.616
Lymphadenopathy, *n* (%)	13 (77)	22 (82)	0.689
KIT D816V EAB in PB, %, median (range)	29 (0-56)	40 (2-80)	0.405
**MARS score at diagnosis**, *n* (%)			
Low-risk, *n* (%)	12 (67)	7 (26)	0.007
Intermediate-risk, *n* (%)	3 (17)	3 (11)	0.591
High-risk, *n* (%)	12 (67)	17 (63)	0.799
**Treatment and outcome**			
Follow-up, years since diagnosis; median (range)	4.0 (1.2-17.0)	2.0 (0.1-12.2)	0.100
Follow-up, years since 1st cycle; median (range)	2.8 (0.8-12.0)	1.1 (0.0-3.8)	0.010
Years to treatment since diagnosis; median (range)	0.8 (0.0-5.0)	0.7 (0.0-11.0)	0.876
Years of treatment duration; median (range)	0.4 (0.1-2.4)	0.3 (0.0-0.6)	0.030
Number of cladribine cycles, median (range)	1.0 (0.5-4.8)	1.0 (0.4-2.5)	0.151
Cycles per months, median (range)	5.5 (1.0-6.0)	3.0 (1.0-8.0)	0.020
Deaths, *n* (%)	10 (56)	20 (71)	0.270
Median OS, years (95% CI)	3.4 (2.9-4.0)	1.5 (1.0-2.0)	0.006

ANC, absolute neutrophil count; ASM, aggressive systemic mastocytosis; BM, bone marrow; CI, confidence interval; EAB, expressed allele burden; MARS, mutation-adjusted risk score; MC, mast cell; MCL±AHN, mast cell leukemia with/without an associated hematologic neoplasm; NR, monocytosis non-response; OS, overall survival; PB, peripheral blood; R, monocytosis response; SM-AHN, systemic mastocytosis with an associated hematological neoplasm.
